# GPs’ perspectives on diagnostic testing in children with persistent non-specific symptoms: a qualitative study

**DOI:** 10.3399/BJGP.2023.0683

**Published:** 2024-10-15

**Authors:** Lianne JW Mulder, Sophie M Ansems, Marjolein Y Berger, Guus CGH Blok, Gea A Holtman

**Affiliations:** Department of Primary and Long-term Care, University Medical Center Groningen, University of Groningen, the Netherlands.; Department of Primary and Long-term Care, University Medical Center Groningen, University of Groningen, the Netherlands.; Department of Primary and Long-term Care, University Medical Center Groningen, University of Groningen, the Netherlands.; Department of Primary and Long-term Care, University Medical Center Groningen, University of Groningen, the Netherlands.; Department of Primary and Long-term Care, University Medical Center Groningen, University of Groningen, the Netherlands.

**Keywords:** children, diagnostic test, general practitioners, primary health care, persistent non-specific symptoms, qualitative research

## Abstract

**Background:**

Diagnostic testing is prevalent among children with persistent non-specific symptoms (PNS), and both undertesting and overtesting have negative consequences for child and society. Research in adults with PNS has shown that GPs use diagnostic testing for reasons other than diagnosis, but comparable research has not, to the best of our knowledge, been conducted in children. Understanding GPs’ perspectives of testing decisions in children could provide insights into mechanisms of undertesting and overtesting.

**Aim:**

To investigate GPs’ perspectives of conducting or refraining from diagnostic testing in children with PNS and the differences compared with their motives when treating adults.

**Design and setting:**

Qualitative study using semi-structured interviews with Dutch GPs.

**Method:**

We purposively sampled GPs until data saturation. Reasons for conducting or refraining from diagnostic tests were explored using two real-life cases from daily practice. Online video interviews were transcribed verbatim. Data were collected and analysed concurrently by thematic content analysis.

**Results:**

Twelve GPs participated. Their decision making involved a complex trade-off among four themes: medical considerations (for example, alarm symptoms), psychosocial factors (for example, doctor–patient relationship), consultation management (for example, ‘quick fix’), and efficient resource utilisation (for example, sustainability). Compared with when treating adults, GPs were more hesitant to conduct diagnostic testing in children because of their higher vulnerability to fearing invasive procedures, lower probability of organic disease, and reduced autonomy.

**Conclusion:**

As in adults, GPs’ decisions to conduct diagnostic tests in children were motivated by reasons beyond diagnostic uncertainty. Educational programmes, interventions, and guidelines that aim to change the testing behaviours of GPs in children with PNS should target these reasons.

## Introduction

Additional diagnostic testing is increasingly prevalent in primary care, especially for the investigation of persistent non-specific symptoms (PNS), in which diagnostic testing ranges between 30% and 59%.^1^^–^^4^ PNS in children include, among others, fatigue, abdominal pain, headache, and musculoskeletal pain.^5^ These symptoms have multiple causes, with a low probability of severe organic disease, and symptoms of organic and functional disorders often overlap.^6^ Although undertesting can result in diagnostic error and delay and undertreatment,^7^^–^^9^ overtesting can lead to false positives, anxiety, patient harm, and overtreatment.^10^^–^^12^ Both are associated with extra costs.^7^^,^^11^ GPs must find a balance between undertesting and overtesting, which could be accomplished by adhering to evidence-based guidelines.^5^^,^^13^ However, the scarcity of guidelines for diagnostic testing in paediatric PNS could make it difficult for GPs to find this balance.

Understanding the motivations of GPs when making decisions about diagnostic testing for PNS could lead to strategies that will improve the current levels of undertesting and overtesting. Studies in adults with PNS indicate that GPs perform tests for various reasons, including for reassurance, improving the doctor–patient relationship, saving time, or simply to be seen doing something.^14^^–^^18^ Reasons to refrain from testing are studied less extensively, but they include the likelihood of background psychosocial factors,^4^ fear of overtreatment,^17^ and trust in a patient’s history and physical examination.^17^ Few studies have specifically examined PNS,^4^^,^^17^ but none, to the best of our knowledge, have looked at PNS in children.^5^ Decisions about diagnostic testing in children might be because of different motivations, such as fear of hurting the child, parental pressure, and different clinical presentations and diagnoses compared with adults.^19^ To date and to the best of our knowledge, no research has investigated GP motivations when considering diagnostic testing in children with PNS.

**Table:** How this fits in

It is known that GPs employ diagnostic tests in adults with persistent non-specific symptoms for motives beyond strictly diagnostic purposes but, to the best of our knowledge, comparable research has not been conducted in children. This study adds that although GPs want to limit un necessary invasive procedures in children, non-diagnostic motives to test are considered important, for example, to provide reassurance or to help maintain the GP–patient relationship. The decision to conduct diagnostic tests in children with persistent non-specific symptoms is based on a complex trade-off among medical considerations, psychosocial factors, consultation management, and efficient resource utilisation. Awareness among GPs of the motives underlying their own testing behaviour in children with PNS could prompt changes in their testing practices.

This study aimed to investigate GP perspectives when considering diagnostic tests in children with PNS and how they differ from considering diagnostic tests in adults. Such knowledge might provide insights to help limit undertesting and overtesting in these children.

## Method

### Study design

We conducted a qualitative study with a deductive–inductive method, integrating pre-existing insights from the literature with new insights derived during analysis.^20^ Data were collected through semi-structured interviews, enabling a thorough exploration of themes.^21^

Reporting is based on the Standards for Reporting Qualitative Research checklist^22^ and Consensus Reporting Items for Studies in Primary Care checklist.^23^

### Responders

GPs or GP registrars who regularly have consultations with children with PNS in the northeast of the Netherlands were recruited between February and May 2023. Emails detailing the study were sent to the research team’s network and to 290 GP practices affiliated with AHON (Academisch Huisarts Ontwikkel Netwerk).^24^ All participants provided digital informed consent. Purposive sampling was performed by sex, years’ experience as a GP, doctorate degree, and the practice location (urban or rural). Interviews focused on encounters with children who report physical complaints that persist beyond a few weeks and that may interfere with daily function (that is, PNS), such as abdominal pain, fatigue, headaches, and musculoskeletal complaints.^22^ Data collection continued until saturation, defined as the moment when interviews yielded no new insights regarding the research questions.^20^

### Data collection

The research team involved a medical doctor and qualitative researcher, two academic GPs, a senior primary care researcher, and a sixth-year medical student. During a brainstorming session, the research team deliberated from scratch what the motivations for GPs could be to conduct or refrain from diagnostic testing, wondered whether the child’s age might play a role, and what the influence of guidelines in this context may be. Using insights from this session, we developed a topic list based on sensitising concepts derived from literature (see Supplementary Information S1); GPs’ perceptions about conducting diagnostic testing (for example, reassurance, exclusion of a diagnosis, improving or maintaining the doctor–patient relationship, diagnostic certainty, and time saving) or refraining from diagnostic testing (for example, fear of causing the child pain or anxiety, and being unlikely to provide reassurance).^14^^–^^16^^,^^19^^,^^25^^–^^28^ We also considered factors specific for children, such as the communication strategies used by GPs (for example, lack of shared decision making and a paternalistic approach) and relevant child-specific factors (for example, fear of quick deterioration, missing serious disease, and hurting or scaring the child).^14^^,^^19^^,^^27^^,^^29^^,^^30^

Using this topic list we developed an interview guide (see Supplementary Information S2), which contained open-ended questions about motives for and against diagnostic testing (defined as diagnostic tests in addition to medical history and physical examination, such as blood and faeces tests or ultrasounds) to further deepen the perspective behind these motives. In addition, the interview guide contained questions about differences between paediatric and adult patient cases and communicating diagnostic tests and their results. To provide a framework for the interviews, GPs were asked in advance to think of two real-life cases from their clinical practice, one in which they conducted and one in which they refrained from diagnostic testing in a child with PNS.

One author conducted all interviews, audio-and video-recording them in Microsoft Teams and transcribing them verbatim. Given the interviewer’s lack of experience, she received guidance and advice from experienced researchers and engaged in multiple practice interviews. The interviewer knew one GP through her work as a GP assistant and one through an internship. With the others she had no prior relationship. Prior to each interview, the interviewer stated her background (medical student), the research objectives, and that all data would be anonymised. Of the 19 invited GPs, seven did not participate either because they no longer practiced as a GP (*n* = 1), they had insufficient availability (*n* = 2), or they gave no reason (*n* = 4).

After 10 interviews, no new answers or insights related to the research questions were provided, so two more GPs were interviewed to confirm data saturation. The average interview duration was 55 min (range 48–74). Quotes were translated from Dutch to English by two authors, edited by a native English speaker, and checked again by the two authors to ensure their meaning remained. All participants received a summary of their interview for a member check, after which two GPs provided minor adjustments (see Supplementary Information S3).

### Analysis

Thematic analysis with a realist approach was used,^31^ with data collected and analysed concurrently. Findings and field notes were discussed during research team meetings after two, six, and 10 interviews to allow emerging themes to be incorporated and explored in subsequent interviews (see Supplementary Information S4). The first three interviews were coded independently by three authors, and discrepancies were discussed to reach consensus. An initial coding framework was developed and one author coded the remaining interviews. These were then reviewed by a second author using the same framework to ensure consistency. The coding framework underwent continuous revision. By the tenth interview, we critically examined the coding framework and started to merge categories, leading to the overarching themes. The final coding and categorisation were discussed in a fourth research team meeting. Atlas. ti 22 was used for data processing and analysis.

## Results

### Responders

Table 1 shows that there is considerable diversity in our purposive sampling criteria for the participating GPs.

**Table 1.** Responder characteristics (*n* = 12)^a^

**Responder ID**	**Sex**	**Age, years**	**Years practising**	**Doctorate degree**	**Practice location**	**GP, type**	**Have children themselves**	**Children in practice, %^b^**
1	Female	36	3	Yes	Rural	Salaried	No	14.7
2	Female	45	14	No	Urban	Practice owner	No	17.8
3	Female	30	0	No	Rural	Locum	No	Unknown
4	Female	37	6	Yes	Urbanised rural area	Locum	Yes	20.0
5	Female	36	7	No	Rural	Locum	Yes	18.8
6	Female	32	0	No	Urbanised rural area	Locum	Yes	Unknown
7	Male	37	6	No	Rural	Locum	No	27.1
8	Male	53	6	No	Urban	Salaried	Yes	19.8
9	Male	54	22	No	Rural	Practice owner	Yes	19.9
10	Female	37	10	Yes	Urbanised rural area	Practice owner	Yes	16.0
11	Male	58	25	No	Rural	Practice owner	Yes	15.2
12	Male	45	16	No	Rural	Practice owner	Yes	24.0

a *As reported by the responders themselves.*

b *Percentage of children aged 0–18 years in the GP practice’s patient population as reported by GP.*

### Themes

We identified four main themes that influenced the motivations of GPs for conducting or refraining from diagnostic testing in children with PNS: medical considerations, psychosocial factors, consultation management, and efficient resource utilisation (Box 1). Decisions about diagnostic testing were described by GPs as a trade-off among multiple factors:

*‘A lot depends on how the conversation goes* [and] *the parents’ concerns … often you prioritise contact with parents, like* [the need for] *their continued trust in the doctor–patient relationship.’*

(GP 6)

**Box 1.** Overview of GP considerations to use or not to use diagnostic testing in children with persistent non-specific symptoms

**Medical considerations**		**Psychosocial factors**	

**Excluding serious disease**	**Scientific evidence**	**Reassurance**	**GP–patient relationship**
+/− (no) Objectifiable abnormalities	+/− Follow guidelines	+/− Reassurance of child/parents	+/− Strong GP–patient relationship
+/− Gut feeling	+/− Diagnostic value	+ Magical effect of diagnostic test	+ Prevent power struggle
+/− (no) Risk factors	– No clinical consequences	– Testing can cause extra anxiety	– Convinced test not necessary with explanation
+/− (no) High impact complaint	**Negative effects**	**Child and parent**	
– Prior negative results	– Medicalisation	+/− (no) Psychosocial determinants	**GP**
	– Harm	+/− (no) Request for testing	+ Little experience
		+/− (no) Child objects to undergoing test	+ Previous abnormal finding
			– GP is also a parent

**Consultation management**			**Efficient resource utilisation**

**Tactical**		**Provide an alternative to diagnostic testing**	+ Avoid medical shopping
+ Quick fix			+/− Costs
– Enough time for explanation		– Monitor (actively)	+/− Sustainability
– Long-term parent education		– Start therapeutic trial	– Waiting list for test
+ Prevent individual harms of more invasive testing or referral		– Give guidance	– Waste of time for child/parents
+ Use low-invasive testing more quickly		– Referral	
+ Standards within GP practice			

*+ = Motives to use diagnostic testing. – = Motives to refrain from diagnostic testing. +/− = Motives for both conducting and refraining from diagnostic testing.*

### Medical considerations

GPs were more inclined to initiate diagnostic testing for indications of possible serious disease, relying on the presence of objective abnormalities during history and examination (for example, abnormal symptom course or alarm symptoms, such as growth restriction) and known risk factors (for example, familial or personal history of illness):

*‘Sometimes there are these keywords … that make you think “That’s odd”. When someone says “He did not grow during the past two years” or “He still has the same clothing size”. Or “He has a round belly and his stool has never been solid”, then you think … “Could it be coeliac disease?”.’*

(GP 5)

Moreover, GPs stated that children were generally less likely to have serious illnesses compared with adults, resulting in less need for diagnostic testing in children:

*‘Well, the likelihood of an adult actually having something abnormal is generally higher than with children, you know. So, I might opt for additional testing sooner in adults.’*

(GP 7)

Gut feeling was important. It might be relied on to consider that something could be wrong, prompting further testing, or could offer reassurance to the GP, making further testing unnecessary:

*‘So, if you have a few of those clues that strike you as odd … a “huh moment” …* [about the clues] *that is not very concrete … that’s usually when, as a general practitioner, you do something extra.’*

(GP 6)

GPs tended to use more diagnostic testing where the complaint had a significant impact, manifesting as school absenteeism, prolonged duration of complaints, and repeat consultations. They expressed reluctance to use diagnostic testing if there was a low suspicion of underlying pathology, a lack of objective abnormalities, or symptoms had little impact. GPs were cautious about repeating previously normal diagnostic testing for the same complaint, and several GPs mentioned refraining from diagnostic testing in children because of potential for harm and medicalisation:

*‘The harm to the child, that’s something I find really important. So, the more we can take a child with a very simple complaint out of a hospital or a medical setting, the better. And perhaps … we simply medicalise a lot. Also, showing the strength of our bodies; the body resolves many issues on its own.’*

(GP 12)

GPs also took scientific evidence into account. Most GPs used guidelines to inform their decisions about diagnostic value, especially if they were inexperienced. Tests with low diagnostic value, such as rheumatoid factor or Lyme disease, were approached with caution. Diagnostic testing was avoided if the result would not alter treatment or if it had been proposed by patients or parents and was considered irrelevant to the complaints:

*‘We shouldn’t cause harm as doctors. I consider unnecessary blood drawing a harm. If it doesn’t contribute to the treatment or diagnosis … then I believe we shouldn’t do it.’*

(GP 8)

GPs were generally more reluctant to use invasive tests in children than in adults, especially in children aged <12 years:

*‘You’re just putting a needle into the child again, so to speak.’*

(GP 9)

### Psychosocial factors

Many GPs mentioned using diagnostic testing to reassure children or their parents, particularly for persistent concerns. They felt like they were more convincing because of diagnostic testing, and this improved parental trust, especially if testing had a high ‘magical value’, such as abdominal ultrasound:

*‘I can very easily make an ultrasound … and it has a high level of magic, right? … and I do exploit that sometimes. And then you can say “Well, there’s nothing there that shouldn’t be there”. That’s not entirely medically accurate, but I do it sometimes.’*

(GP 9)

By contrast, other GPs emphasised that diagnostic testing was not the ‘holy grail’ and did not necessarily improve symptoms or provide reassurance. It was also mentioned that diagnostic testing could cause extra anxiety, especially if communication between the doctor, parent, and child was not sufficiently transparent, or if the child was afraid of the diagnostic test (for example, drawing blood):

*‘It can reinforce worry if the doctor feels the need to draw blood again; but, that’s not the case. The doctor didn’t find it necessary, the parents did.’*

(GP 8)

Navigating this three-way relationship between child, parent, and physician sometimes complicated the GPs’ decision-making processes, especially when the desires of the child and parent diverged. Although GPs could speak with adults directly, interactions with children often occurred through their parents. Because parents often requested diagnostic testing, GPs saw themselves as defenders of the child, protecting them from unnecessary investigations:

*‘A child essentially doesn’t ask for it themselves, whereas an adult often does. So, with a child, you always have to try to do what’s best for them and the parent. For a parent, that sometimes means blood tests, but not for the child. So, you try to find a middle ground, because a child doesn’t ask to undergo all of that themselves.’*

(GP 3)

Considerations were given to the fact that children lack autonomy to make medical decisions or can be anxious to undergo a diagnostic test, whereas adults can make these decisions for themselves. Consequently, GPs indicated the need for greater caution with children.

GPs tended to use diagnostic testing more in the absence of a psychosocial explanation for the symptoms, and less if there was a possible psychosocial explanation (for example, unstable family situation, school problems, and/or significant anxiety or stress). If parents or children requested a specific diagnostic test, GPs sometimes agreed to avoid discussion and a ‘power struggle’, preferring not to waste effort or strain the doctor–patient relationship:

*‘I’ve always approached it in a way where I don’t want to engage in a power struggle with patients. If they really want it and I don’t think it’s necessary, I can say that … but if people still want it, personally, I don’t think it’s right to say “no, you can’t have it”.’*

(GP 2)

Using communication techniques (for example, verbal judo), GPs also reported that they could convince patients and parents that a diagnostic test was unnecessary, especially when parents or patients expressed low concern, were open to alternative explanations, and trusted the GP:

*‘*[About verbal judo] *If you say “You were talking about taking blood. But as far as I’m concerned, that’s really not necessary medically, because I am reassured”. Usually they are actually very satisfied.’*

(GP 1)

GPs noted that children, often not having specific expectations, were more easily convinced not to undergo diagnostic testing compared with their parents or with adults who have similar complaints.

Diagnostic testing could be avoided if there was a strong doctor–patient relationship, but was sometimes used to build or maintain that relationship and facilitate ongoing patient care:

*‘Then the negotiation begins. If it turns out that they … demand it, and that it’s truly necessary for reassurance, for us to move forward as patient and doctor with that child, then yes, I would do it.’*

(GP 8)

Factors related to GPs also played a role in the decision to test. For example, some young GPs mentioned that they found themselves using diagnostic testing more often because of their lack of experience and the inherent uncertainty surrounding PNS. In addition, the GPs’ previous experiences, such as a previous patient with an unexpected serious condition, could result in more diagnostic testing:

*‘Maybe I was triggered because I saw a young woman a year ago of* [whom] *I thought: “Do I feel an enlarged liver?” I decided to request an ultrasound and she turned out to have a liver tumour.’*

(GP 1)

GPs who were parents themselves also reported greater empathy and communication skill use when interacting with children and their parents. This contributed to a more effective normalisation of complaints and, as some GPs suggested, occasionally decreased the need for diagnostic testing:

*‘My son is very afraid of needles, like very afraid. So it does influence me because I would not want my son to undergo unnecessary testing.* […] *Since I became* [a] *mother myself I have a different view on diagnostic investigations I think.’*

(GP 5)

### Consultation management

GPs occasionally used diagnostic testing for their own convenience (that is, a ‘quick fix’), stating that performing the test is sometimes less time consuming than explaining why the test is unnecessary, especially when the child or parents are not receptive to this explanation:

*‘It is also a quick fix, right? Then you’re quickly done with your consultation and they get what they want … then you think, “well, you do you”.’*

(GP 10)

Circumstantial factors, like less GP motivation after a long workday, also played a role in opting for diagnostic testing. However, GPs who had 20-min appointment slots in their practice (as opposed to the standard 10 min) mentioned that this allowed them more time to explain and discuss matters with patients. GPs felt like this could sometimes prevent diagnostic testing.

In some instances, GPs used low-invasive diagnostic testing (for example, urine or faeces) to avoid the harms associated with more invasive procedures or referrals:

*‘For example, faecal calprotectin. I use that a bit to reassure and avoid having to do blood tests, which I find a bit invasive for children.’*

(GP 6)

GPs often suggested alternatives to diagnostic testing, such as ‘watchful waiting’, a repeat consultation, or recommending a therapeutic trial. Sometimes, the alternative to testing was immediate referral to a paediatrician, who could offer additional diagnostic or treatment options. Lastly, one GP mentioned adjusting their testing standards to meet with the expectations of parents and children in a new practice:

*‘My colleague, the practice owner, has a low threshold for medical intervention. The patients are kind of used to that, you can tell.* [...] *So I have a lower threshold as well; I have adjusted to the practice standards.’*

(GP 4)

### Efficient resource utilisation

To limit healthcare costs, GPs sometimes used less expensive diagnostic tests to prevent more expensive diagnostic tests (for example, magnetic resonance imaging) or prevent patients from seeking care elsewhere:

*‘If I order CRP* [C-reactive protein]*, ESR* [erythrocyte sedimentation rate]*, and leucocytes, and thereby avoid an ultrasound or CAT* [computerised axial tomography] *scan, I am more cost-effective than if I don’t do any blood tests and simply say, “Okay, let’s refer?” So, you have to navigate a bit.’*

(GP 12)

Sustainability was also a motive for GPs to limit diagnostic testing:

*‘We’re currently very focused on sustainability; that is, what is efficient care? This involves both cost-effectiveness and sustainability.* […] *Sometimes doing nothing is better … eliminating what isn’t necessary, including medications, additional diagnostic tests, and travel.’*

(GP 4)

Furthermore, a GP mentioned being cautious when referring for diagnostic tests that have long waiting lists, considering it necessary to reduce the burden on health care and retain availability for patients who really needed it. The waste of patient time and increased costs (especially in rural areas where travel times are longer) was also considered in decisions about diagnostic testing.

## Discussion

### Summary

This study revealed that for GPs, decisions about diagnostic testing involved a complex trade-off between social interactions with the child and their parents; interconnection between specific medical, psychosocial, and consultation management considerations; and efficient resource utilisation. Most GPs expressed a desire to be more hesitant with diagnostic tests for children because of their higher vulnerability to fearing invasive procedures, lower probability of organic disease, and reduced autonomy compared with adults.

### Strengths and limitations

Our findings should be interpreted in the context of its strengths and limitations. First, a strength of this study is that we requested GPs to prepare two real-life patient cases from their own practice, one case each where diagnostic testing was and was not requested for children with PNS. This ensured that the data were grounded in daily practice, allowing for in-depth exploration of the considerations in each patient’s case. Second, three researchers participated in the coding and another two researchers participated in the analysis and interpretation of the findings, thereby offering different perspectives. Third, although the interviewer was inexperienced, we not only provided specific training and practice in the requisite skills but also noted good interview length and depth that gave rise to relevant themes that answered our research questions.

An important limitation is that we could not limit the possibility of cognitive bias or GPs presenting an idealised version of how they ‘should’ have acted; nevertheless, most GPs were open about sharing uncertainties and did not appear to be defensive. GPs appeared to face greater challenges in reducing overtesting than undertesting. However, this could be subject to recall bias, as it might be easier for GPs to remember cases with than without diagnostic testing.

Another limitation is the potential bias from the brainstorming and analysis sessions. The medical background and belief in evidence-based medical decisions of all researchers could have led to a predominantly medical and scientific perspective. In addition, although the participating GPs provided a diverse sample, our interviews were limited to GPs from the north-east of the Netherlands. This will limit the transferability of our findings given that considerations will vary not only by region and country but also by healthcare system. Finally, it is a limitation that we did not collect the cultural background of GPs and their practice populations, and this may influence test-ordering behaviour.^32^

### Comparison with existing literature

To the best of our knowledge, this is the first study to have explored GPs’ motives when making decisions about diagnostic testing in children. GPs expressed a desire to protect children from unnecessary invasive testing, considering them more vulnerable to fearing invasive procedures and having reduced autonomy compared with adults. Indeed, painful diagnostic procedures in children may result in exaggerated memories of the pain and heighten distress during subsequent procedures.^33^ GPs in our study also felt a duty to prioritise the needs of children over the requests of parents, and they avoided diagnostic tests in children because of concerns about medicalisation and their lower chance of organic disease.

Nevertheless, GPs were also motivated to use testing in children for reasons other than for diagnostic confirmation or uncertainty. This complexity, and the influence of non-diagnostic motives on GPs’ decision making, was described by van der Weijden *et al*^17^ in 2002 and confirmed in later studies.^15^^,^^16^^,^^18^ Over the past 20 years, research has provided new insights into the effect of testing for these reasons. For example, there is no evidence that a negative test result provides reassurance;^26^^,^^27^^,^^34^ in fact, it might increase uncertainty, anxiety, and feelings of dismissal or invalidation.^14^ Questionnaire and qualitative studies have also shown that most patients want an explanation and emotional support, not tests.^14^^,^^35^^–^^37^ If physicians still want to use diagnostic tests to achieve reassurance, providing information about the meaning of normal results before testing can improve reassurance.^27^

Communication and time seem to be of crucial influence. GPs in our study expressed that they would refrain from diagnostic testing when they had more time and when they could convince the parents and child that testing was unnecessary. Indeed, research has shown that immediate or delayed test ordering do not influence patient satisfaction or anxiety after a consultation, whereas specific aspects of physician–patient communication do.^37^ A large international randomised controlled trial showed that C-reactive protein testing actually increased antibiotic prescribing for lower respiratory tract infections in primary care, whereas communication training led to decreased antibiotic prescribing.^38^ Our findings show that GPs tried to use diagnostic tests to prevent further medical interventions (such as prescriptions) that might lead to harm or unnecessary costs. GPs should be aware, however, that false- and true-positive results might contradict their intentions and lead to overdiagnosis, overtreatment, and avoidable costs.^10^^–^^12^

We acknowledge that in the context of societal changes, wherein patients are increasingly unaccustomed to uncertainty or apparent inaction and seek medical explanations for symptoms that fall within normal physiological variation, GPs may be more inclined to conduct diagnostic procedures.^39^ Therefore, patients should also be informed about the drawbacks, meaning, and interpretation of tests as this could also reduce the use of low-value tests.^40^ This could be accomplished through decision aids, as is already done for prostate-specific antigen testing,^41^ or patient information leaflets, as is done for vitamin D and B12 testing in primary care.^42^

### Implications for research and practice

Our research shows that GPs may be more hesitant to use diagnostic tests in children with PNS than in adults, but that tests still often fulfil many non-diagnostic roles. Although GPs should certainly be aware that diagnostic tests are designed to reduce diagnostic uncertainty, with the potential for negative consequences when testing for other reasons, it would be naive to conclude that testing for non-diagnostic reasons is always inappropriate. Instead, this study shows that diagnostic tests are an integral part of the GPs’ clinical armoury, with GPs often making a careful and deliberate trade-off between medical need, psychosocial factors, consultation management, and using resources efficiently. Objectives within these latter three domains may be just as beneficial to the overall health of the child, even when the goals could probably be achieved through other methods, such as effective communication.

The complex trade-off between these disparate motivations, the scarcity of evidence-based testing guidelines, and the current societal context of consumerism and medicalisation make it difficult for GPs to adhere to evidence-based testing practices. However, it is beyond dispute that undertesting and overtesting should be limited wherever possible to ensure cost-effective care that meets the needs of the individual patient and wider society. Educational programmes, interventions, and guidelines that seek to change GPs’ testing behaviour could take characteristics of GPs into account. For instance, GPs with greater experience can have more trust in their gut feeling; parental status may empower GP–parents to mitigate unnecessary testing through improved communication; and increased awareness of sustainability and environmental issues could prevent GPs from testing. To facilitate this behavioural change, patients should be informed about the drawbacks, meaning, and interpretation of tests alongside GPs.
